# Long-Term Home-Use of Sensory-Motor-Integrated Bidirectional Bionic Prosthetic Arms Promotes Functional, Perceptual, and Cognitive Changes

**DOI:** 10.3389/fnins.2020.00120

**Published:** 2020-02-19

**Authors:** Jonathon S. Schofield, Courtney E. Shell, Dylan T. Beckler, Zachary C. Thumser, Paul D. Marasco

**Affiliations:** ^1^Department of Mechanical and Aerospace Engineering, University of California, Davis, Davis, CA, United States; ^2^Department of Biomedical Engineering, Lerner Research Institute-Cleveland Clinic, Cleveland, OH, United States; ^3^Advanced Platform Technology Center, Louis Stokes Cleveland VA Medical Center, Cleveland, OH, United States; ^4^Research Service, Louis Stokes Cleveland VA Medical Center, Cleveland, OH, United States

**Keywords:** perceptual engineering, sensory restoration, take-home trial, human-machine interface, prosthesis

## Abstract

Cutaneous sensation is vital to controlling our hands and upper limbs. It helps close the motor control loop by informing adjustments of grasping forces during object manipulations and provides much of the information the brain requires to perceive our limbs as a part of our bodies. This sensory information is absent to upper-limb prosthesis users. Although robotic prostheses are becoming increasingly sophisticated, the absence of feedback imposes a reliance on open-loop control and limits the functional potential as an integrated part of the body. Experimental systems to restore physiologically relevant sensory information to prosthesis users are beginning to emerge. However, the impact of their long-term use on functional abilities, body image, and neural adaptation processes remains unclear. Understanding these effects is essential to transition sensate prostheses from sophisticated assistive tools to integrated replacement limbs. We recruited three participants with high-level upper-limb amputation who previously received targeted reinnervation surgery. Each participant was fit with a neural-machine-interface prosthesis that allowed participants to operate their device by thinking about moving their missing limb. Additionally, we fit a sensory feedback system that allowed participants to experience touch to the prosthesis as touch on their missing limb. All three participants performed a long-term take-home trial. Two participants used their neural-machine-interface systems with touch feedback and one control participant used his prescribed, insensate prosthesis. A series of functional outcome metrics and psychophysical evaluations were performed using sensate neural-machine-interface prostheses before and after the take-home period to capture changes in functional abilities, limb embodiment, and neural adaptation. Our results demonstrated that the relationship between users and sensate neural-machine-interface prostheses is dynamic and changes with long-term use. The presence of touch sensation had a near-immediate impact on how the users operated their prostheses. In the multiple independent measures of users’ functional abilities employed, we observed a spectrum of performance changes following long-term use. Furthermore, after the take-home period, participants more appropriately integrated their prostheses into their body images and psychophysical tests provided strong evidence that neural and cortical adaptation occurred.

## Introduction

The human hand is extremely versatile, capable of performing tasks with remarkable variations in the required dexterity, power, and precision of grasps. These range from tasks as delicate as microsurgeries to those as demanding as rock climbing. Cutaneous sensation is vital to controlling our hands and upper limbs. In nearly every activity performed with our hands, cutaneous sensation shapes how we achieve that task. Specifically, it closes the motor control loop by informing the real-time adjustments of grasping forces and responses to perturbations during object manipulations (Johansson, 1996). Cutaneous sensation also plays a critical role beyond limb control by providing much of the necessary information the brain requires to perceive our limbs as a part of our bodies (embodiment) (Botvinick and Cohen, 1998), which helps us distinguish ourselves as separate from the world around us.

Prosthesis solutions have become increasingly sophisticated and advanced robotic limbs are beginning to rival healthy limbs in dexterity (Belter et al., 2013). Unfortunately, the increased sophistication of these devices reveals that the lack of natural sensory feedback and reliance on open-loop control limits the functional potential of these devices. Humans naturally seek to close the loop through sensory information. This can be seen clearly in prosthesis users who typically adopt indirect feedback strategies in an effort to compensate for the lack of sensation. This involves continual visual attention paid to prostheses and monitoring of other indirect cues such as the sound of the motors, vibrations, and changes in pressure or leverage between the prosthetic socket and the residual limb (Gonzalez et al., 2012; Schofield et al., 2014). This substituted sensory information is cognitively demanding to interpret and can leave users feeling overwhelmed and frustrated (Gonzalez et al., 2012). Addressing the challenges associated with the absence of sensation is a highly active field of study, and attempts to provide prosthesis users with sensory feedback have been reported as early as the 1950s (Siehlow, 1951). More recently, the use of mechanotactile and vibrotactile feedback has been used to provide sensations of proportional tactile force (Marasco et al., 2011; Antfolk et al., 2013; Rombokas et al., 2013; Cipriani et al., 2014; Hebert et al., 2014; De Nunzio et al., 2017), and movement sensation (Sharma et al., 2014; Witteveen et al., 2014; Hasson and Manczurowsky, 2015; Marasco et al., 2018) in both amputee and able-bodied populations. These methods have proven effective in patient performance of tasks such as precise force generation (De Nunzio et al., 2017), force discrimination (Hebert et al., 2014), stiffness discrimination (Hebert et al., 2014; Witteveen et al., 2014), stimuli localization (Antfolk et al., 2013), and multi-site sensory discrimination (Antfolk et al., 2013). Other approaches are also being pursued, including electrical stimulation of peripheral nerves (e.g., Christie et al., 2017), electrocutaneous stimulation (e.g., Paredes et al., 2015), and direct cortical stimulation of the primary somatosensory cortex (e.g., Tabot et al., 2013; Hiremath et al., 2017).

When a limb is lost, there is a disruption of one’s body image (Rybarczyk and Behel, 2008), which is likely potentiated by the absence of sensory feedback (Marasco et al., 2011). The perception that our limbs belong to our bodies is largely a product of visual and tactile information; when touch to a body part is seen and felt appropriately, our brains assume ownership over that body part (Botvinick and Cohen, 1998). Therefore, the absence of sensation in upper-limb prostheses significantly impedes these devices from being perceived as integrated parts of the body. When taken together, operating an insensate prosthesis leaves the user to pilot a numb, cognitively demanding, and disconnected tool rather than an integrated replacement limb. Although no commercially available prostheses actively provide physiologically relevant sensory feedback, efforts to achieve intuitive touch feedback, among other sensory modalities, are on the experimental horizon.

The implications of sensory loss extend far beyond the direct impediments to prosthesis use and the disruptions to body image. Amputation damages all the nerves that once connected to the limb, which promotes structural and functional reorganization of sensory-motor pathways (Cohen et al., 1991; Flor et al., 1995; Makin et al., 2013). Regular prosthesis use appears to have an important influence on how the brain adapts to limb loss. There is evidence to suggest that the regularity and the extent to which one uses a conventional mechatronic (myoelectric) prosthesis correlates with reduction in this cortical reorganization (Lotze et al., 1999) and may even influence the network of brain areas from which body schema and representation are processed (Boccia et al., 2019).

Although conventional insensate prosthesis use may have a long-term influence on cortical adaptation, it is important to make the distinction that these devices still do not leverage the same residual neural pathways that the intact limb once did. Communicating with the user and brain via these same mechanisms is perhaps the most direct way to truly replace a limb. Taking advantage of existing circuitry that the body and user are pre-wired to accept can enable intuitive control, physiologically relevant feedback, and rapid incorporation as part of the body. In recent years, there has been an emergence of surgical interventions that interface and communicate with the residual neural anatomy of a limb post-amputation. For example, targeted motor reinnervation and targeted sensory reinnervation [TMR and TSR, respectively (Kuiken et al., 2004; Hebert et al., 2014)] are surgical techniques that create motor and sensory neural-machine-interfaces (NMIs) for intuitive closed-loop control of mechatronic prostheses. These procedures surgically redirect motor and sensory nerves, which once served the patient’s amputated hand, to proximal muscle and cutaneous sites in the residual limb (Kuiken et al., 2004; Hebert et al., 2014). When a patient attempts to move their missing limb, the reinnervated muscle sites will contract. This muscle activity can be measured and used to control mechatronic prosthesis movements (Kuiken et al., 2004). Furthermore, cutaneous stimulation of the reinnervated skin sites is experienced as occurring on the missing limb (Kuiken et al., 2007a). Patients can experience sensations of touch, force, vibration, temperature, and pain in the missing limb with near-normal detection thresholds (Kuiken et al., 2007a). By instrumenting a prosthetic limb to detect touch and force, and mapping these signals to touch feedback devices located on a patient’s reinnervated skin sites, participants can experience touch and grasp forces of a prosthesis as though it is their missing limb (Hebert et al., 2014).

With a newly restored sense of touch, TSR participants have demonstrated improvements in functional tasks requiring the ability to detect prosthetic digit touch and discriminate forces (Hebert et al., 2014). Furthermore, psychophysical and metabolic evidence suggests that TSR participants using a touch feedback interface receive the appropriate sensory information to begin re-embodying artificial limbs (Marasco et al., 2011). Imaging data suggest that reinnervated participants attempting to activate a prosthetic hand produce similar activation in the primary motor cortex as healthy controls, which was not the case with a non-reinnervated participant group (Serino et al., 2017). Similarly, touch on the reinnervated skin activated the primary sensory cortex in patterns similar to those of a healthy control group, although activation strength was reduced (Serino et al., 2017). Taken together, it is evident that TMR-TSR participants operating an NMI mechatronic prosthesis are equipped with all the necessary pieces to operate and feel an artificial limb as though it were an integrated part of the body. However, NMI prostheses are still machines that must communicate with the user. Although the neural mechanisms of this communication are native to the user, the relationship is likely dynamic over time as the user learns to optimally interact with their device, and the brain adapts to the newly restored sensory-motor channels. Performing long-term take-home trials with sensate NMI prostheses can help us understand how users learn, embody, and adapt to these systems. This is an important next step to unlocking artificial limbs that are truly reintegrated and functional beyond the laboratory.

We recruited TMR-TSR participants to perform long-term take-home trials of touch-integrated NMI robotic prostheses. Participants completed assessments before and after the take-home period that captured changes in functional ability, prosthesis embodiment, as well as cognitive changes. We hypothesized that following the take-home period, we would see indications of limb reintegration in the form of improved functional outcomes, increased scores on prosthesis embodiment surveys, and changes in psychophysical-cognitive tests.

## Materials and Methods

### Take-Home Study Structure

Prior to the take-home period, we benchmarked the performance of each participant on a series of experiments with their NMI prosthesis, with various touch conditions, described in a later subsection. Participants repeated these experiments after the take-home period. The experiments completed were touch mapping of reinnervated skin (Kuiken et al., 2007a), a temporal order judgment task [TOJ; (Marasco et al., 2011)], a block-foraging stiffness discrimination task (Beckler et al., 2019), a psychophysical Fitts’ law grasp force task (Thumser et al., 2018), the Box and Block task (Mathiowetz et al., 1985), and the Clothespin Relocation task (Miller et al., 2008). These experiments are briefly described below and additional procedural details are available in the references cited above. After baseline performance was assessed in their initial visit, participants completed a minimum 9-month take-home period. SD and TH completed this take-home period with their sensate NMI prostheses while CTRL completed this period with his normal (insensate) TMR-controlled NMI myoelectric prosthesis. During this take-home period, all participants logged their prosthesis use and completed a diary describing activities performed with their prostheses.

### Participants and Technical Setup

**FIGURE 1:**
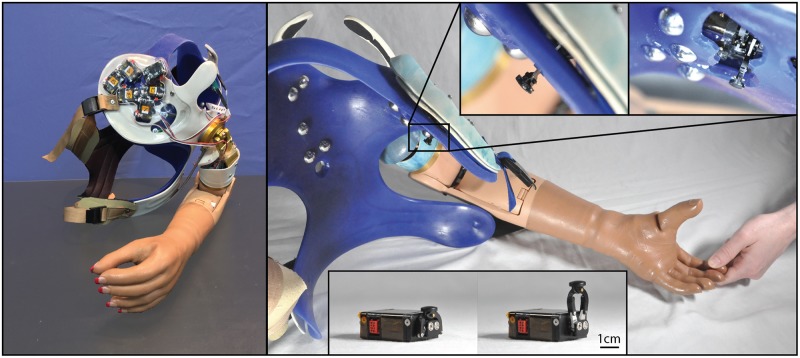
Photographs of the closed-loop NMI prosthesis system created for and used by the participant with a shoulder disarticulation (SD). Touch tactors placed in the chest plate are shown in the left panel, while dome electrodes and bump switches used for control are shown in the right main panel. Touch tactors extended when sensors embedded in the prosthetic hand detected touch. Close-up views of the touch tactors extended are shown in the top inset, and an unmounted tactor in the rest and extended position is shown in the bottom inset.

This study was carried out under a protocol approved by the Institutional Review Boards of the Cleveland Clinic and Department of the Navy Human Research Protection Program. Participants gave written informed consent prior to study procedures. All participants had previously undergone both TMR and TSR surgeries following amputation and trained to use a myoelectric prosthesis system with their reinnervated muscles. All participants perceived touch on the reinnervated skin of their residual limbs (touch sites) as touch on their missing hand. We created a closed-loop NMI prosthesis for each participant. A certified prosthetist fitted a new myoelectric prosthesis system, using components comparable to their familiar, prescribed system. We added touch feedback by placing robotic, four-bar haptic pushing devices (touch tactors) on the reinnervated skin at their touch sites (HDT Global, Fredericksburg, VA, United States) (Kim et al., 2010). Photographs of one participant’s prosthesis are shown in Figure 1. Tactor activation was mapped to matching sensorized locations on the prosthetic hand, thereby translating touch on the prosthesis to touch on the missing hand at the corresponding location. Details of each participant’s NMI prosthesis are described below.

To detect touch on the prosthesis, we retrofitted the first, second, and third digits of a SensorHand Speed (Ottobock, Duderstadt, Germany) with strain gauges, and the palm and fourth and fifth digits of a System Inner hand shell (Ottobock, Duderstadt, Germany) with force-sensitive resistors. Sensors were paired with tactors so that when touch was detected by one of the six sensors in the prosthetic hand, a tactor pushed on the site where participants perceived touch on their missing hand corresponding to the activated sensor.

The sensors and tactors were configured to simultaneously apply two different touch feedback modes – proportional touch and tap detection. The proportional touch mode mapped the amount of force generated on the sensorized prosthetic hand to force applied by the tactors. The tap detection mode relayed the sensation of object contact or tapping to the missing hand by causing the tactors to quickly and forcefully extend based on the speed and amplitude of the force applied to the sensorized prosthesis, and then rapidly retract. Together, the proportional mode continuously dictated the end position of the tactor, while contact events and other transient forces applied an additional, brief extension of the tactors, which then rapidly returned to the proportional mode command. To tune touch feedback forces, participants watched an investigator touch their prosthetic hand as well as touched it with their intact hand; tactor force gains (mapping forces on the prosthesis to touch force feedback) were adjusted until the participants reported satisfaction with the subjective experience and were able to uniquely identify touch on each digit. Both the proportional and tap modes could be tuned independently, and while this may be a slight departure from the way an intact individual experiences touch and force sensation, it allowed participants to be sensitive to light touches which, in a pure proportional mode, might fail to make tactor-skin contact. In practice a combination of both was preferred for most digits by the participants.

The completed NMI prostheses were self-contained and required no extra work from the participants to don/doff and maintain beyond the requirements of a standard myoelectric prosthesis. The touch feedback system drew power from the same battery used to power the myoelectric prosthesis, so the participants only had to charge the standard battery for a Boston Digital Arm Systems Elbow (LTI/Liberating Technologies, Inc., Holliston, MA, United States) to ensure power for the entire system. Furthermore, the touch feedback system was fully integrated, with no external components. The sensors were integrated into the terminal device and the cosmesis, with no visible or protruding pieces. The touch tactors were integrated into the socket and shrouded to protect them from damage and streamline appearance. This integration also meant that the feedback system was placed using the repeatability of socket donning. To use the touch-enabled NMI, participants simply donned their prosthesis as normal; no additional technical knowledge or training was necessary.

#### Participant With a Shoulder Disarticulation, TMR, and TSR

The first participant, SD, had previously received targeted motor and sensory reinnervations and regularly used a left shoulder disarticulation, socket-fit myoelectric prosthesis system with proportional EMG control (Kuiken et al., 2007a, b; Marasco et al., 2011). For the take-home period and experimental testing, SD used a myoelectric prosthesis system comparable to her familiar, prescribed system. The prosthesis used a custom (Advanced Arm Dynamics, Redondo Beach, CA, United States) silicone-lined, electrode-embedded socket with chest plate, harness, and dropped shoulder, a Boston Digital Arm Systems Elbow, a SensorHand Speed set to speed 0, System Inner hand shell, and proportional EMG control. This system afforded her three active simultaneous degrees-of-freedom – elbow flexion/extension, wrist pronation/supination, and hand open/close – as well as three passive degrees-of-freedom – shoulder flexion/extension, shoulder abduction/adduction, and humeral rotation. We located six touch sites, one each on all five digits and her palm, where touch on the reinnervated skin of the residual limb site caused sensation of touch on her missing hand. All of the touch sites were located on the skin over her pectoral muscle, so we mounted six four-bar, linear-actuating tactors to the prosthesis chest plate, positioned over her touch sites. Thus, pressure on the prosthetic hand caused SD to perceive congruent touch sensation on her missing hand. This system provided distinct touch sensation in physiologically correct locations for her five digits and palm.

#### Participant With a Transhumeral Amputation, TMR, and TSR

The second participant, TH, had previously received targeted motor and sensory reinnervations, and regularly used a left transhumeral myoelectric prosthesis system with a socket liner, controlled by pattern recognition (Dumanian et al., 2009; Marasco et al., 2011, 2018). For this study, TH used a comparable prosthesis system, consisting of a custom-made thermoplastic socket and harness, electrode-embedded liner (The Ohio Willow Wood Company, Mt. Sterling, OH, United States), Boston Digital Arm Systems Elbow, SensorHand Speed set to speed 0, System Inner hand shell, and pattern recognition control (CoAPT, Chicago, IL, United States). This system afforded her three active degrees-of-freedom – elbow flexion/extension, wrist pronation/supination, and hand open/close – as well as passive humeral rotation. Tactors extended through holes drilled in the socket and pushed on TH’s touch sites through thinned areas of the liner. This system provided distinct touch sensation in physiologically correct locations for her five digits and palm.

#### Control Participant With a Transhumeral Amputation, TMR, and TSR

The third participant, CTRL, had previously received targeted motor and sensory reinnervations, and regularly used a left suction-socket, myoelectric prosthesis system with proportional EMG control (Hebert et al., 2014; Marasco et al., 2018). For testing, CTRL used a comparable prosthesis system, consisting of a custom-made, electrode-embedded thermoplastic socket and harness, Boston Digital Arm Systems Elbow, SensorHand Speed set to speed 0, System Inner hand shell, and proportional EMG control. This system afforded him two active simultaneous degrees-of-freedom – elbow flexion/extension and hand open/close – as well as passive humeral rotation and wrist pronation/supination. CTRL had two touch sites, one where touch on the residual limb site caused sensations of touch on his thumb and index finger, and the other sensation of touch primarily on his index finger (and faint middle finger). Therefore, two sensors and two tactors were configured, the first sensitive to touch on the prosthetic thumb and the second to the index finger. Prior to testing, CTRL reported distinct touch sensation in physiologically correct locations for both tactors. CTRL elected to have his tactors configured to operate only on proportional mode as this was subjectively more appropriate to him. Although CTRL was fitted with a sensate prosthesis for experiments in the lab, he did not take this touch-enabled prosthetic limb home during the take-home period. He instead used his own non-touch, TMR-controlled myoelectric arm as his home-use limb.

### Touch Conditions

During the experiments described below, participants completed tasks with the touch tactors configured in four different ways (touch conditions). In the *touch-off* condition, the tactors were powered off, and no touch feedback was provided. In the *touch-on* condition, participants received spatially and temporally congruent touch feedback. In the *lagged* condition, the tactors provided feedback after a 1000-ms delay, i.e., spatially congruent but temporally incongruent. In the *scrambled* condition, the sensor-tactor mapping was pseudo-randomized such that activating a sensor caused a mismatched tactor to actuate, so touch feedback was spatially incongruent but temporally congruent with touches on the prosthetic hand. Touch conditions were completed in blocks. Note that during CTRL’s visits the lagged and scrambled conditions were omitted from relevant tests when needed to accommodate time constraints related to the participant’s work schedule and international travel.

### Experiments

#### Touch Mapping

##### Experimental procedure

To determine where to place touch tactors, we queried participants about the locations at which they experienced touch on their missing hand when we stimulated reinnervated skin. We also repeated this process after the take-home period to assess any changes in touch sensation locations. For all participants, we prepared for touch mapping by identifying reproducible sites on their reinnervated skin to be tested for touch sensation. We used skin-based landmarks, such as scars or freckles, and where possible, we used a thermoplastic reference socket with a 1 × 1 cm grid of holes drilled through it. We passed a felt marker through the holes to consistently draw the 1 × 1 cm alphanumeric grid. This ensured that we could interrogate the same locations before and after the take-home period. We applied pressure to each point in a randomized order with a cotton swab, which was attached to a 300 g Von Frey monofilament to ensure equal pressure was applied to each point of the grid. Participants were given schematic diagrams of hands that they drew on to indicate where they felt touch sensation on their missing hand [percept drawings (Kuiken et al., 2007a)]. It should be noted that following TMR-TSR procedures, touch on reinnervated skin is felt only in the missing hand (Kuiken et al., 2007a; Hebert et al., 2014). If participants felt touch on only their native skin (i.e., upper arm or chest), they were instructed to inform the investigator but not to draw anything as this indicated that the location touched was not reinnervated. This procedure was performed twice for each participant: once before and then again following the take-home period. Also note that CTRL’s initial touch map was created with the cotton swab placed on a 300 g Von Frey monofilament. However, instead of using a reference grid, the point placement was guided by the points shown in Hebert et al. (2016) (a study in which he was previously involved). There were fewer points represented in Hebert et al. (2016) because that earlier study used a thresholding approach to define touch areas. In CTRL’s final touch map session this change in point referencing methodology was corrected and the mapping procedure was conducted in alignment with the mapping procedure for all participants as described above.

##### Data analysis

We digitally transcribed each participant’s percept drawings and layered all points onto two representative schematics (touch maps) for each participant, one per visit. Transparencies of individual percept drawings were normalized across participants and visits such that equal shades of color indicated that an equal proportion of tested points caused sensation in that region. For each participant’s touch mapping session, we first looked for a change in proportion of touch map locations, for which the participant reported sensation, using a standard *z*-test. Then, we divided their hand drawings coarsely into twelve regions (five individual digits, plus the remainder of the hand/palm, for both ventral and dorsal surfaces) and compared shifts in the proportion of sensation reported in each of the different locations for each participant, comparing their initial to final visit. This analysis of proportions was designed to ensure validity by allowing for differences in the amount/exact location of tested points in the initial and final maps.

We also wanted to isolate the areas of reinnervated skin that were targeted by the touch tactors to understand how perceptions of touch at these areas might change when stimulated long-term. This analysis was performed *post hoc* using experimental photographs to identify where each touch tactor was located relative to the alphanumeric points used in the touch mapping experiments. Since the exact points at which tactors contacted the skin may have varied slightly day-to-day due to normal differences in donning, we also included the percept drawings from tested points adjacent to the tactor locations when compiling touch maps for these areas (with shading normalized as described above).

#### Temporal Order Judgment Task

##### Experimental setup

This task assessed relative weighting of sensory processing between the intact and amputated sides by asking the participants to judge which of two nearly simultaneous events, one on each side, occurred first. Participants were seated at a table across from an investigator, with a partition placed in between them. While their view of each other was occluded, a small window in the partition allowed the participant and investigator to interact. Participants placed their prosthetic hand within reach of the investigator through the window and could view their prosthesis as it was touched by the investigator. Each participant had a commercially available vibratory unit (C2 tactor, Engineering Acoustics Inc., Casselberry, FL, United States) taped to their skin near the distal end of their residual limb, and another vibratory unit in a mirrored position on their intact limb. For each participant, we placed two foot pedals under the table, near their feet. During the experiment, participants wore disposable earplugs and noise-canceling headphones that played gray noise.

##### Experimental procedure

Participants were instructed to watch their prosthetic hand at all times during the experiment, unless it was covered, in which case they watched a small marker placed on the partition just above the prosthetic hand. Each experimental trial started and ended with a 1-min rest period where the prosthetic hand was covered with a white sheet. After the initial rest period, the white sheet was removed, and the seated investigator repeatedly touched the prosthesis in different randomized locations in an experimental protocol similar to Marasco et al. (2011). After 5 min of stimulation, the vibratory units placed on the left and right sides activated asynchronously, with the delay between activation of the unit on one side and activation of the unit of the other side varying (10, 20, 30, 60, 90, or 120 ms), while hand stimulation continued. Half of the time vibration occurred on the left side first, and the other half of the time right-side vibration was first. Participants were instructed to decide (two-alternative forced choice) which unit vibrated first, and to press the foot pedal on the side that vibrated first. Foot pedal presses were recorded. Time interval and left-first/right-first order was pseudo-randomized in sets of 12 presentations, with each combination of time interval and left-first/right-first occurring once per set. One experimental trial contained seven sets, for a total of 84 presentations. Blocks of testing consisted of five trials: the four touch conditions (touch-off, touch-on, lagged, and scrambled) and a fixation trial (where the prosthetic hand remained covered and the investigator did not interact with the prosthetic hand). Within each experimental block, the order of conditions was randomized. Participants completed three blocks of testing.

After each TOJ trial was completed (each touch condition), participants were given a nine-statement, seven-point Likert scale survey to measure the degree to which they embodied the prosthetic hand (Marasco et al., 2011). Participants were asked to indicate their level of agreement with each statement, from *strongly disagree* to *strongly agree*. Of the nine statements, three were related to embodiment, and the remaining six were used to control for suggestibility and task compliance.

Note that SD participated in two initial visits (before the take-home period). The temporal order judgment data were collected on the first visit, while the remaining data were collected in the second visit upon receiving a satisfactorily fitting prosthesis that she could wear over the time course of the take-home period.

##### Data analysis

For each participant, we calculated a *point of subjective simultaneity* (PSS) (Keetels and Vroomen, 2012). We first calculated the proportion of left-first/right-first responses for each time interval presented (12 in total, described above). A sigmoid was then fit across these 12 proportions. The time interval for which left-first and right-first responses were modeled as being equal was taken as the PSS.

Embodiment was calculated by averaging questionnaire statements following each TOJ trial. The three statements of embodiment were averaged and the six control statements were averaged for each participant according to each touch condition (touch-off, touch-on, lagged, scrambled, and fixation). Participants were considered to have embodied the prosthesis for a particular touch condition if their average response was greater than or equal to one (Kalckert and Ehrsson, 2012).

#### Block-Foraging Stiffness Discrimination Task

##### Experimental setup

The block-foraging stiffness discrimination task is a scientifically validated sensory-motor function test that was specifically designed to be sensitive to touch feedback. This is achieved through quantifying performance during selection of rubber blocks with a target stiffness from a pool of target and distractor blocks (Beckler et al., 2019). This task provides separable assessment of motor and sensory performance as well as insight into strategy. Following the procedure described by Beckler et al. (2019), we placed rubber blocks (25.4 mm cubes) of varying stiffness in the testing area (approximately 500 mm long and 400 mm wide, with 3 mm walls to contain the blocks) on a table in front of the participants. There were 60 blocks total, 20 “hard blocks” of 80A durometer, 20 “medium blocks” of 60A durometer, and 20 “soft blocks” of 40A durometer. One reference block of each hardness was labeled and placed outside the box, within participants’ reach. To reduce auditory cues, participants wore disposable earplugs and noise-canceling headphones playing gray noise. Participants wore frosted lenses that mitigated discrimination by visual cues, but still allowed them to visually locate the blocks. Two video recorders were used to film the experiment from different angles.

##### Experimental procedure

Participants were informed that the testing area contained soft, medium, and hard blocks, and that they would be searching for either soft or hard blocks. We instructed the participants which hardness to search for by tapping the corresponding reference block. Participants tapped the target reference block to indicate they were starting the trial. They then searched for and removed five blocks, one at a time, from the testing area that they thought were the target hardness. We recorded the number of blocks the participants squeezed, the number of correct blocks the participants selected, and trial duration. Participants also performed baseline trials where they were instructed to select five blocks of any hardness and move them outside the testing area. Soft, hard, and baseline trial order was randomized and completed in blocks of about 20% of the entire task. Blocks of testing alternated between touch-off and touch-on conditions. For each touch condition, participants selected 100 rubber blocks total.

##### Data analysis

We divided the trial durations into three different sub-sections: *search time*, the time it took a participant to find the block they ultimately selected; *involvement time*, the time a participant spent discriminating block stiffness and transporting the block they ultimately selected; and *handling time*, the time a participant spent transporting a block during the baseline trials (where no discrimination was made). We also calculated *recognition time*, the time a participant spent making their discrimination decision when selecting a block, as the difference between *involvement time* and *handling time*. Time values were derived through frame-by-frame analysis of video footage captured at 30 frames per second.

We calculated each participant’s *accuracy* by dividing the number of correct blocks that were selected by the total number of blocks selected. We used their accuracy, the total number of blocks they encountered, and the known proportions of blocks to calculate a *false positive rate* (the probability a participant incorrectly selected a non-target block when they encountered one) and a *false negative rate* (the probability a participant incorrectly rejected a target block when they encountered one) for each participant.

*Efficiency*, calculated by dividing accuracy by the average time it took to select a block, provided an overall performance measure. *Discrimination efficiency*, calculated by dividing accuracy by average recognition time, provided a discrimination performance measure.

To determine if changes across touch condition and visit were statistically significant, *z*-tests of proportion were used for accuracy, Wilcoxon rank-sum tests were used for search times, and *t*-tests were used for recognition and handling times. A significance level of 0.05 was used for all tests.

#### Psychophysical Fitts’ Law Grasp Force Task

##### Experimental setup

The psychophysical Fitts’ law grasp force task is a scientifically validated sensory-motor function test developed to quantify the user’s ability to quickly and accurately produce a desired grasping force (Downey et al., 2018; Thumser et al., 2018). This speaks to participants’ abilities to incorporate sensory feedback into their control scheme. Each participant sat at a table in front of a television screen with their prosthetic hand in reach of a grip force manipulandum. A partition was used to block the participant’s view of the manipulandum. A keyboard was placed within reach of the participant’s healthy hand. Participants were instructed to rest their open prosthetic hand around the manipulandum, but not to touch it between trials. An investigator monitored the prosthetic hand to ensure task compliance.

##### Experimental procedure

Before the experiment began, we asked each participant to squeeze the manipulandum with the full force of their prosthesis to record the maximum grip force, analogous to their maximum voluntary contraction. Their maximum force was measured three times, and the average was considered their maximum prosthesis grip force.

During the task, participants watched the television screen and were shown an image of a familiar, everyday item (e.g., apple, wine glass, milk carton, or eggshell). When they were ready to start the trial, they pressed the keyboard spacebar, which initiated a red-yellow-green “traffic light” countdown. When the green light lit, an audible tone played, and the participant squeezed the manipulandum with the force needed to pick up the displayed object without dropping or damaging it. We instructed the participants to grasp the manipulandum as quickly and accurately as possible. When the participant achieved their desired grip force, they pressed the spacebar again to end the trial. We recorded the maximum force generated by the participant, and the time elapsed from force onset until the maximum force was reached. Participants were shown eight unique items, and the order of items was randomized. With items that had multiple grasping possibilities, such as the wine glass, we asked participants to choose one single way that they imagined they would grasp that object and consistently use that imagined grasp every time they saw the object. The task was completed in blocks of 32 trials, and blocks alternated between touch-off and touch-on conditions. Participants were shown each unique item a total of 20 times per condition, for a total of 160 trials per touch condition.

During TH’s initial visit we noticed that she prioritized speed over precision, hindering the ability to identify her maximum grasp force precision. To capture this for future participants and sessions, we added a “precision” version of the task, in which the instructions were identical, except participants were shown the same object (milk carton) every trial, and they were told to be as accurate as possible, but that speed was not important. The precision block was 20 trials long per touch condition.

##### Data analysis

Consistent with Thumser et al. (2018), we calculated three outcome measures from the grasp force task: *objects successfully handled*, *peak precision*, and *speed*. Adopting their definitions for this test, an object was considered *successfully handled* if it was statistically different from the participant’s maximum prosthesis force. Successful handling purposefully does not include information about object-damage threshold or force required to lift an object; rather, it focuses the metric on a participant’s ability to repeatably achieve their intention, without grading them on their knowledge of object durability or weight. Each object’s target difficulty was determined from the ratio of average force amplitude to force variability. *Peak precision* was the maximum difficulty of successful objects [calculated as maximum effective index of difficulty (IDe) in units of bits]. Each participant’s “*speed*” was determined as the average ratio of target difficulty to average trial duration for all successful objects (calculated as *throughput* in units of bits per second). Throughput describes the tradeoff between speed and precision, where a higher value indicates that the user does not slow down much as task difficulty increases, and low values indicate that difficult tasks result in a dramatic reduction in speed. This is in notable contrast to the participant being fast or slow in absolute terms, which is not described by this metric.

For all participants except TH in her initial visit, the peak precision measured during the “precision” version of the task replaces the peak precision if it is higher than what was produced during the primary task.

#### Box and Block and Clothespin Relocation Tasks

##### Experimental setup

Standard tasks to characterize manual dexterity were performed to assess motor control. Both of these tasks involve manipulating and relocating small objects under time constraints and are used in clinical practice but were developed prior to the clinical availability of sensory feedback in prostheses. For the Box and Block task, a standard two-compartment Box and Block box (Mathiowetz et al., 1985) with center partition was placed on a table in front of the participant. One side of the box was filled with 25.4 mm wooden cubes and placed on the same side as the participant’s prosthesis. For the Clothespin Relocation task, we placed a standard Clothespin Relocation setup (Miller et al., 2008) on a table in front of the participant. The setup included a horizontal bar, with three clothespins positioned equidistantly, and a vertical bar. For both tests, we quantified eye gaze patterns as a proxy for the visual attention required to complete each task. The participant wore an eye-tracking headset (ETL500, ISCAN, Woburn, MA, United States) that automatically tracked gaze in space as well as detected and tracked the participant’s hand relative to their gaze vector by color-based object detection of a brightly colored glove worn on the prosthetic hand during the task. In half of the trials we also employed a visual distractor. A laptop placed just beyond the box or clothespin setup played a distractor video, which showed three blocks that were randomly moved on and off screen and periodically prompted the participant to report how many blocks were shown on screen.

##### Experimental procedure

For the Box and Block task, participants started with their prosthetic hand on the table and were given a “3, 2, 1” countdown to begin the trial, then had 60 s to move as many blocks as they could from the filled compartment, over the center partition, to the other compartment. Participants were instructed to move only one block at a time; if multiple blocks were moved, only one was counted. At the end of the 60-s period, the number of blocks correctly transferred was recorded. For the Clothespin Relocation task, participants were instructed to move each clothespin from the horizontal bar to the vertical bar, one at a time without dropping them. After the three clothespins were successfully transferred, they were reset equally spaced on the vertical bar, and participants transferred them back down to the horizontal bar. If a clothespin was dropped, the trial was reset to the last completed transfer. Successfully transferring the clothespins to and from the vertical bar concluded a single trial. Both the time taken to move the clothespins up and down were recorded. For both tasks, each block of testing contained four trials of different touch conditions: touch-off, touch-on, lagged, and scrambled. Each trial was completed twice, first with and then without the visual distractor. Participants completed three blocks, and within each block the touch condition order was randomized. Participants also completed three trials using their intact limb to assess their able-bodied level of performance in those tasks.

##### Data analysis

Box and Block scores for each touch condition were calculated as the average number of blocks successfully transferred in 60 s across the three trials. Clothespin Relocation scores for each touch condition were calculated as the average time needed to successfully transfer three clothespins to the vertical bar and back to the horizontal bar, across the three trials. Failed trials (i.e., when a clothespin was dropped) were not included in the time calculation, although the number of failed trials per condition was recorded.

To quantify visual attention paid to each participant’s hand, we calculated the root-mean-squared (RMS) gaze deviation, which is the angular difference between their gaze vector and the center of their hand, less the average radius of the detected hand area (to a minimum difference of zero). Thus, a higher RMS gaze deviation, in degrees, represents more time spent looking farther away from their hand, and a lower RMS gaze deviation represents more time looking at or near their hand.

## Results

### Take-Home Period

SD had her touch-enabled arm for 2 years and we received activity diaries for 25 weeks. During the 2-month period immediately before her final visit, she wore the arm for an average of 4.9 ± 3.7 h per week. TH also had her touch-enabled arm for 2 years and we received activity diaries for 17 weeks. She wore her arm an average of 5.6 ± 1.1 h per week in the 2-month period prior to her final visit. CTRL wore his regularly prescribed (insensate), TMR-controlled NMI myoelectric arm during his workday until the battery drained, typically 9–10 h. Over the course of one and a half years, he provided activity diaries for 40 weeks. During this time, for 22 weeks in which he wore his myoelectric arm and reported wear time, he wore it an average of 5 ± 2 days per week.

Both of the participants who used the take-home system were unilateral amputees and felt that they could perform their jobs and day-to-day activities without their prostheses, conventional or sensate. TH reported needing her prosthesis for various tasks around her house (e.g., laundry, cutting vegetables, opening items) but that it was hard to wear for long periods of time due to eventual discomfort and the weight of the device. SD reported that she did not feel that she needed her prosthesis for many tasks, other than some use in preparing meals and eating. Both SD and TH avoided wearing their prosthetic arm system if there was a possibility that it might get wet (e.g., rain, going to the beach, participating in aquatic activities), and both lived in areas and had hobbies where this occurred frequently. In contrast, CTRL has a physically demanding job that requires carrying multiple, large, and/or bulky items. As a result, he wore his arm for long periods of time throughout his workday unless in wet, muddy, or extremely cold conditions.

### Touch Mapping

**FIGURE 2:**
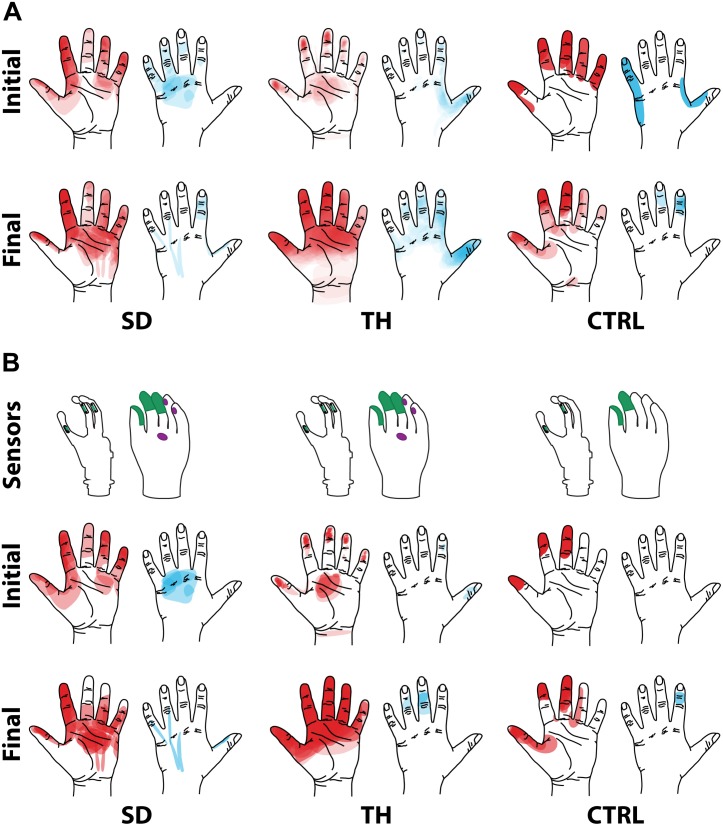
Regions of the hand where the participant with a shoulder disarticulation (SD), the participant with a transhumeral amputation (TH), and the control participant (CTRL) felt sensation when their reinnervated skin was touched in the initial visit and final visit are shown. Red shading indicates where sensation was felt on the ventral side of their hand, and blue shading indicates where sensation was felt on the dorsal side of their hand. The intensity of the shaded areas indicates the proportion of probed locations on the reinnervated skin for which sensation was perceived at that location on the missing hand. **(A)** Shows sensations reported from all points tested on each individual participant’s reinnervated skin. **(B)** Shows the reported sensations arising from the reinnervated skin near/surrounding the tactor locations. The areas of each participant’s prosthetic hand that were instrumented are shown in the top row. The locations of the strain gauges in the digits of the SensorHand Speed are shown in green on the schematic of the prosthetic hand. The regions where the sensors responded are shown on the cosmesis in green for the strain gauges and purple for the force sensitive resistors.

**FIGURE 3:**
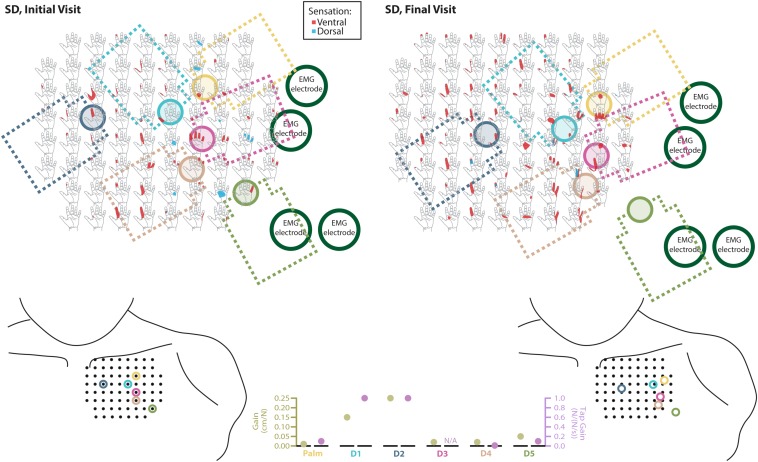
Responses to touch mapping for the participant with a shoulder disarticulation (SD). The initial visit is shown on the left while the final visit is shown on the right. Sensations that were reported as projected to the ventral or dorsal side of the missing hand are shown in red and blue, respectively. The locations of the tactor heads that pushed on SD’s skin are depicted as shaded circles and the tactor motor bodies are outlined with dotted lines; these correspond to sensors located on the prosthesis palm (yellow), thumb (D1, light blue) index finger (D2, medium blue), middle finger (D3, magenta), ring finger (D4, tan), and little finger (D5, light green). Locations of EMG electrodes near the reinnervated skin are portrayed as dark green circles. The line drawings below the maps for each visit depict the locations of the points mapped and the tactor heads (colored circles) on SD’s upper chest. The proportional (gold markers) and tap (mauve markers) gains for each of the six tactors are shown in the panel in the bottom center.

**FIGURE 4:**
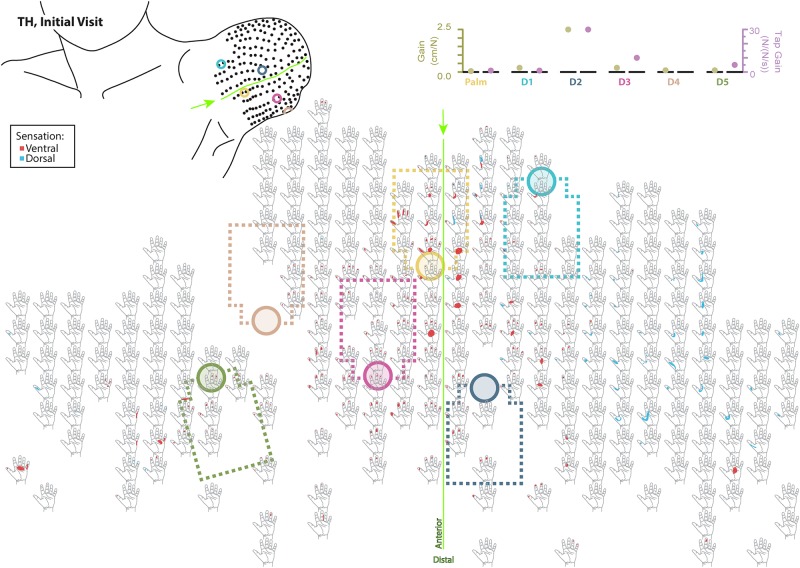
Initial responses to touch mapping for the participant with a transhumeral amputation (TH). Sensations that were reported as projected to the ventral or dorsal side of the missing hand are shown in red and blue, respectively. The locations of the tactor heads that pushed on TH’s skin are depicted as shaded circles and the tactor motor bodies are outlined with dotted lines; these correspond to sensors located on the prosthesis palm (yellow), thumb (D1, light blue) index finger (D2, medium blue), middle finger (D3, magenta), ring finger (D4, tan), and little finger (D5, light green). The line drawing on the upper left depicts the locations of the points mapped and the tactor heads (colored circles) on TH’s upper arm. The proportional (gold markers) and tap (mauve markers) gains for each of the six tactors are shown in the panel in the top right. Bright green lines and arrows are provided to help orient the reader between the points drawn on the residual limb and the maps. Note that the wider spacing of the maps from the distal end of the residual limb is a result of projecting the surface of the three-dimensional residual limb onto a two-dimensional page; touch mapping points were uniformly spaced around the residual limb. Also note EMG control electrode locations are not depicted as this participant used a pattern recognition EMG control system and silicone liner. Therefore, effective control is relatively insensitive to electrode location, and electrode position may vary with respect to tactors with each donning.

**FIGURE 5:**
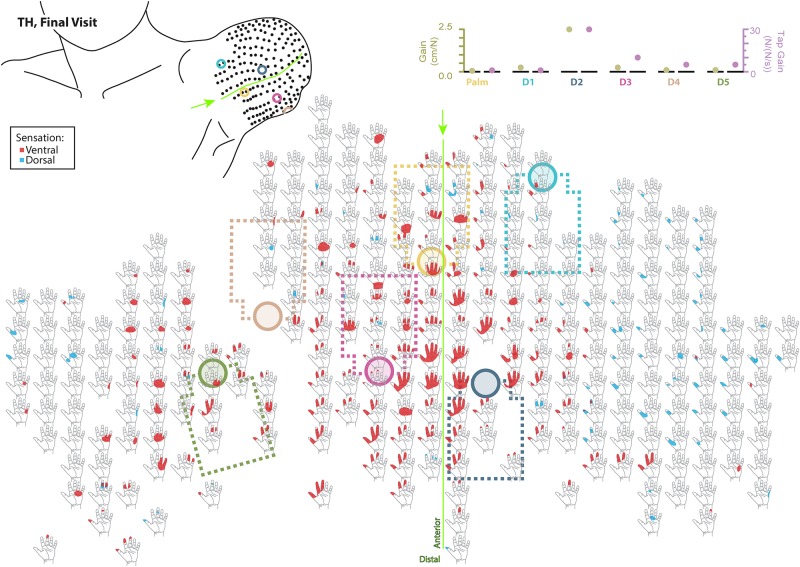
Final responses to touch mapping for the participant with a transhumeral amputation (TH). Sensations that were reported as projected to the ventral or dorsal side of the missing hand are shown in red and blue, respectively. The locations of the tactor heads that pushed on TH’s skin are depicted as shaded circles and the tactor motor bodies are outlined with dotted lines; these correspond to sensors located on the prosthesis palm (yellow), thumb (D1, light blue) index finger (D2, medium blue), middle finger (D3, magenta), ring finger (D4, tan), and little finger (D5, light green). The line drawing on the upper left depicts the locations of the points mapped and the tactor heads (colored circles) on TH’s upper arm. The proportional (gold markers) and tap (mauve markers) gains for each of the six tactors are shown in the panel in the top right. Bright green lines and arrows are provided to help orient the reader between the points drawn on the residual limb and the maps. Note that the wider spacing of the maps from the distal end of the residual limb is a result of projecting the surface of the three-dimensional residual limb onto a two-dimensional page; touch mapping points were uniformly spaced around the residual limb. Also note EMG control electrode locations are not depicted as this participant used a pattern recognition EMG control system and silicone liner. Therefore, effective control is relatively insensitive to electrode location, and electrode position may vary with respect to tactors with each donning.

**FIGURE 6:**
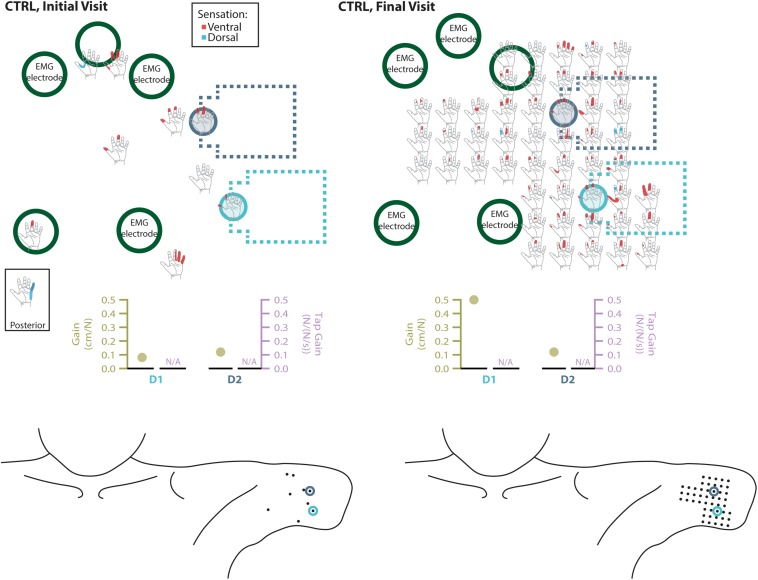
Responses to touch mapping for the control participant (CTRL). The initial visit is shown on the left while the final visit is shown on the right. Sensations that were reported as projected to the ventral or dorsal side of the missing hand are shown in red and blue, respectively. The locations of the tactor heads that pushed on CTRL’s skin are depicted as shaded circles and the tactor motor bodies are outlined with dotted lines; these correspond to sensors located on the prosthesis thumb (D1, light blue) and index finger (D2, medium blue). Locations of EMG electrodes near the reinnervated skin are portrayed as dark green circles. The line drawings below the maps for each visit depict the locations of the points mapped and the tactor heads (colored circles) on CTRL’s upper arm. The proportional (gold markers) and tap (mauve markers) gains for each of the two tactors are shown below the maps. Note that one point was probed on the posterior side of the residual limb; the map showing the response for that point is shown in the box labeled ‘Posterior.’ Also note, while the number of tested points varied between initial and final mapping sessions, the points tested in the final mapping session were an expanded data set. This expansion captured the sensations reported over the same area of reinnervated skin but with greater resolution. Subsequent analyses based on data from these points (Figure 2) were designed to mathematically accommodate this methodological difference. All points tested are shown, regardless of whether or not the participant reported feeling sensation projected to their missing hand when touched at that location.

Both SD and TH reported a significantly greater proportion of touch-sensitive locations on areas tested on their reinnervated skin in their visit after the take-home period compared to their initial visit, while CTRL did not (SD: 91.7 vs. 79.2%, *p* = 0.034; TH: 79.8 vs. 59.1%, *p* < 0.0001; CTRL: 93.9 vs. 90.0%, *p* = 0.66; *z*-test). The increase in SD’s reported sensations comes primarily from her ventral thumb and palm; TH’s increase is concentrated in the ventral ring finger, index finger, and palm, and on the dorsal index and middle fingers (Figures 2–5). There was a high degree of spatial congruency between the instrumented regions on the prosthetic hand and the corresponding percepts on the missing hand (Figure 2B). From the initial to final visit: TH demonstrated an increase in the amount of missing hand represented at tactor-stimulated areas; SD demonstrated an increase in how much of the palm and index finger was felt, a focusing of the area represented on the thumb, and a decrease in the area reported for the ventral hand; CTRL demonstrated relatively minimal changes (Figure 2B). Figures 3–6 highlight the individual points tested on each participant’s reinnervated skin and the reported sensations projected to the missing hand, with tactor placements and control electrodes overlaid. Together these results suggest that with regular stimulation of the reinnervated skin, the representation of each participant’s missing hand expanded and strengthened over time.

### PSS

**FIGURE 7:**
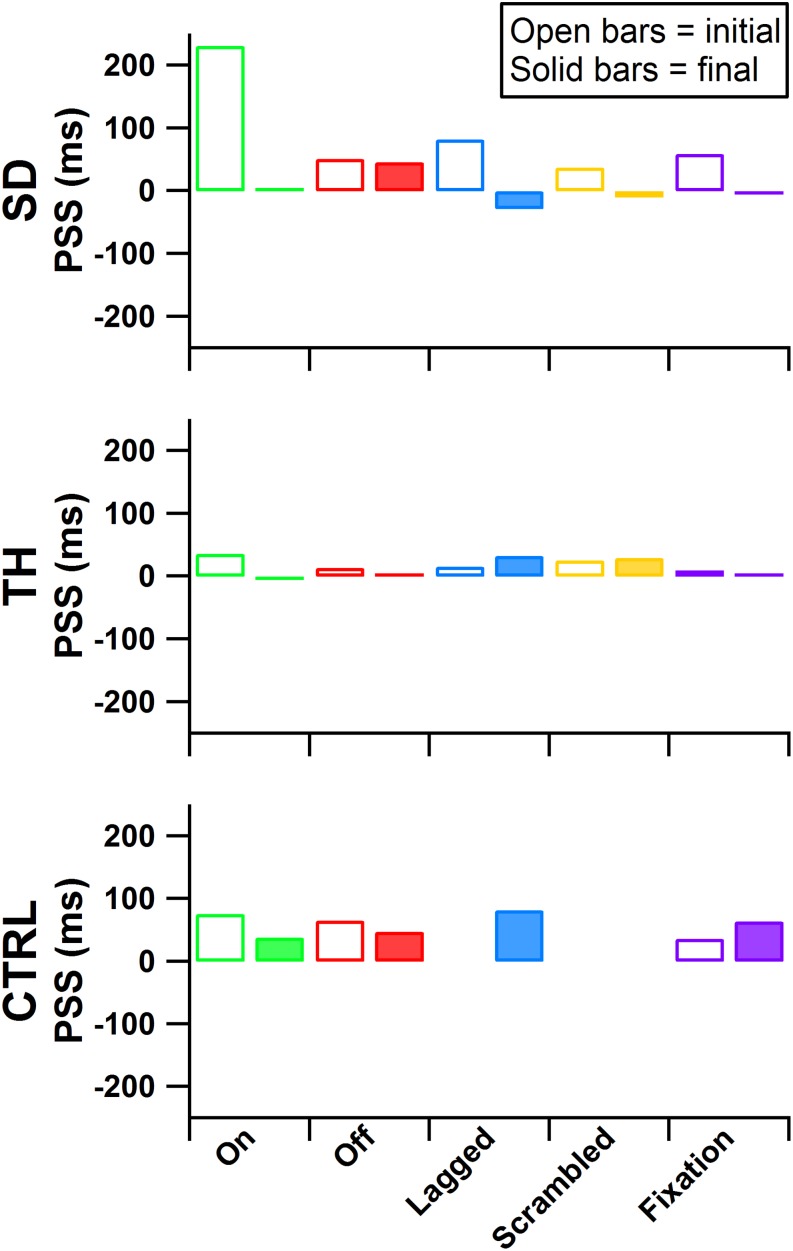
Point of subjective simultaneity (PSS) is presented for the participant with a shoulder disarticulation (SD, top row), the participant with a transhumeral amputation (TH, center row), and the control participant (CTRL, bottom row) for their visits before (open bars) and after (filled bars) the take-home period. From left to right, participants’ results from the touch-on (green), touch-off (red), lagged (blue), scrambled (yellow), and fixation (purple) conditions are shown. Values further from zero indicate greater asymmetry in the PSS between the intact and amputated sides.

During the initial visit, all participants demonstrated asymmetries in the point of subjective simultaneity (PSS) across all touch conditions (Figure 7, average PSS across all conditions SD: 92 ms, TH: 20 ms, CTRL: 59 ms), suggesting that participants’ brains weighted sensory information from the amputated side asymmetrically from the intact side. In the final visit SD demonstrated symmetry in PSS scores (average PSS across all conditions 1 ms). TH also demonstrated reduced PSS bias (average PSS across all conditions 12 ms). The remaining 12 ms difference was entirely due to the lagged and scrambled conditions (32 and 29 ms, respectively), whereas the other three conditions had an average PSS of 0 ms. CTRL again demonstrated asymmetric PSS during the final visit (average PSS across all conditions was 58 ms). The decreased PSS asymmetry for SD and TH, but not CTRL, suggest that extended exposure to touch feedback led to more comparable processing of sensory information from the two sides.

### Embodiment Questionnaires

**FIGURE 8:**
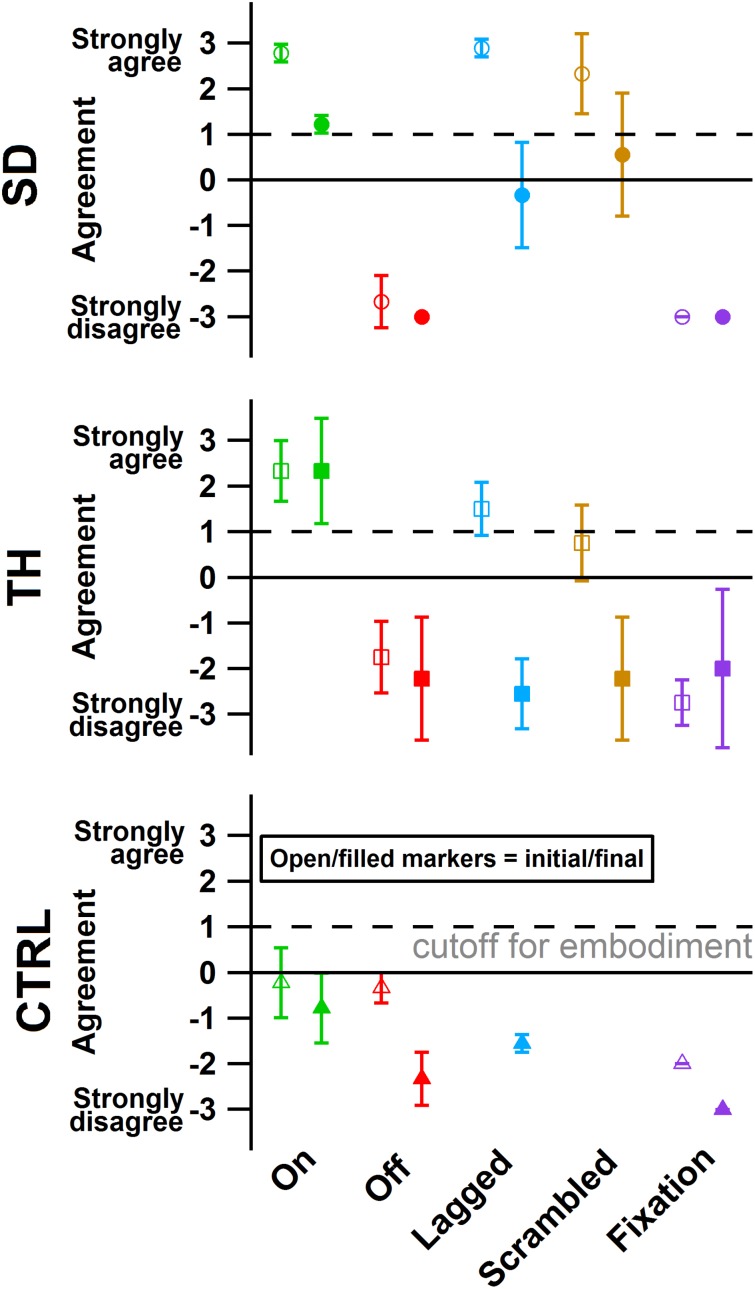
The degree of agreement with questionnaire statements is shown for the participant with a shoulder disarticulation (SD, top row, circles), the participant with a transhumeral amputation (TH, center row, squares), and the control participant (CTRL, bottom row, triangles) in their visit before (open markers) and after (filled markers) the take-home period. Responses to control questions are provided in Supplementary Figure 1. All participants responded below the cutoff for agreement to the control questions. Markers are colored according to touch feedback condition: touch-on (green), touch-off (red), lagged (blue), scrambled (yellow), and fixation (purple). Participants were considered to have embodied their prosthesis if their agreement with embodiment questions was greater than or equal to one (Kalckert and Ehrsson, 2012), indicated by the dashed line.

During the initial visit, SD’s and TH’s survey responses indicated that they embodied their prostheses in the touch-on condition. They also both embodied their prostheses in the lagged condition (Figure 8). In the scrambled condition, SD embodied her prosthesis while TH approached embodiment. During the final visit, SD and TH both indicated embodiment for the touch-on condition but no longer embodied the lagged and scrambled touch conditions. CTRL did not indicate embodiment for any condition during the initial or final visit. All participants responded below the cutoff for agreement to the control questions, indicating that their embodiment scores were not due to participant suggestibility (Supplementary Figure 1).

### Block-Foraging Stiffness Discrimination Task

**FIGURE 9:**
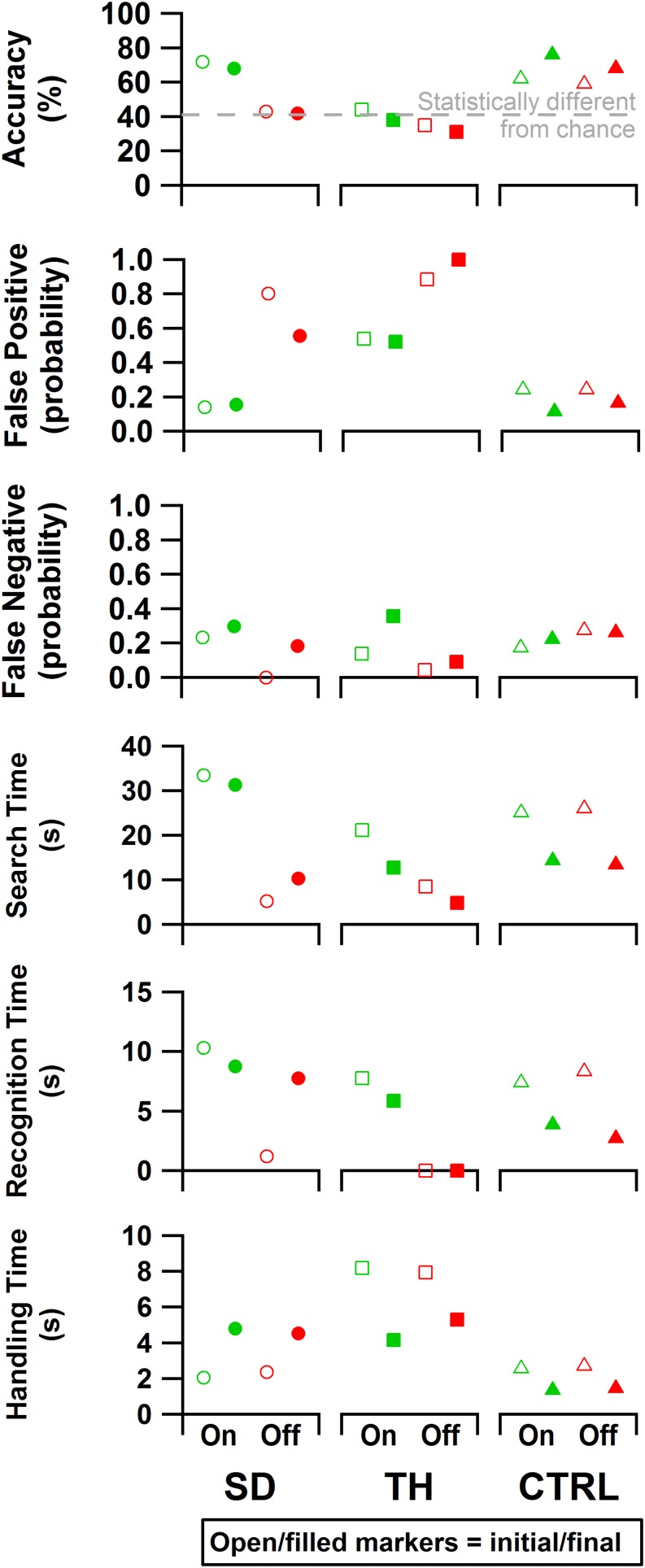
From top to bottom: accuracy, false positive rate, false negative rate, search time, recognition time, and handling time data are presented. Results are shown for the participant with a shoulder disarticulation (SD, left column, circles), the participant with a transhumeral amputation (TH, center column, squares), and the control participant (CTRL, right column, triangles) in their visit before (open markers) and after (filled markers) the take-home period. Data are presented for the touch-on (green) and touch-off (red) conditions. The gray dashed line in the accuracy plot indicates the smallest statistically detectable change (41%) from chance accuracy (33%).

Both during the initial and final visits, all participants demonstrated an increase in discrimination ability when given touch (Figure 9). SD demonstrated an improvement in accuracy that was statistically significant during the initial and final visits (*p* < 0.00001 and *p* = 0.00022, respectively). During TH’s initial visit, providing touch sensation allowed her to achieve an accuracy score that was statistically different from chance, whereas without touch sensation she was unable to discriminate the blocks. This effect was not observed in the final visit. When using touch, SD and TH demonstrated decreases in false positive errors with increases in false negative errors, indicating that participants were more selective.

Both SD and TH showed significant changes in the time spent searching for blocks when touch was turned on (*p* < 0.00001), both during the initial and final visits (Figure 9). CTRL did not demonstrate any significant changes in search time behavior during either visit. During the initial visit, SD and TH showed significantly increased recognition time when touch was turned on (*p* < 0.00001). This may indicate that the participants slowed down to engage with the sensory feedback to help inform decisions. This effect was not present in CTRL. During the final visit, however, TH and CTRL showed significantly increased recognition time when touch was turned on (*p* < 0.00001 and *p* = 0.00035, respectively), whereas SD did not. During both initial and final visits, TH had recognition times that were not significantly different from zero when touch was turned off (*p* = 0.75 and *p* = 0.89, respectively), indicating that without touch feedback discrimination decisions were not attempted. No participant demonstrated any significant changes to handling time during either visit when touch was turned on. However, comparing initial visit to final, TH and CTRL demonstrated significantly faster handling times (*p* < 0.00007), whereas SD had significantly slower handling times (*p* < 0.00001). Efficiency and discrimination efficiency consistent with Beckler et al. (2019) are presented in Supplementary Figure 2.

### Psychophysical Fitts’ Law Grasp Force Task

**FIGURE 10:**
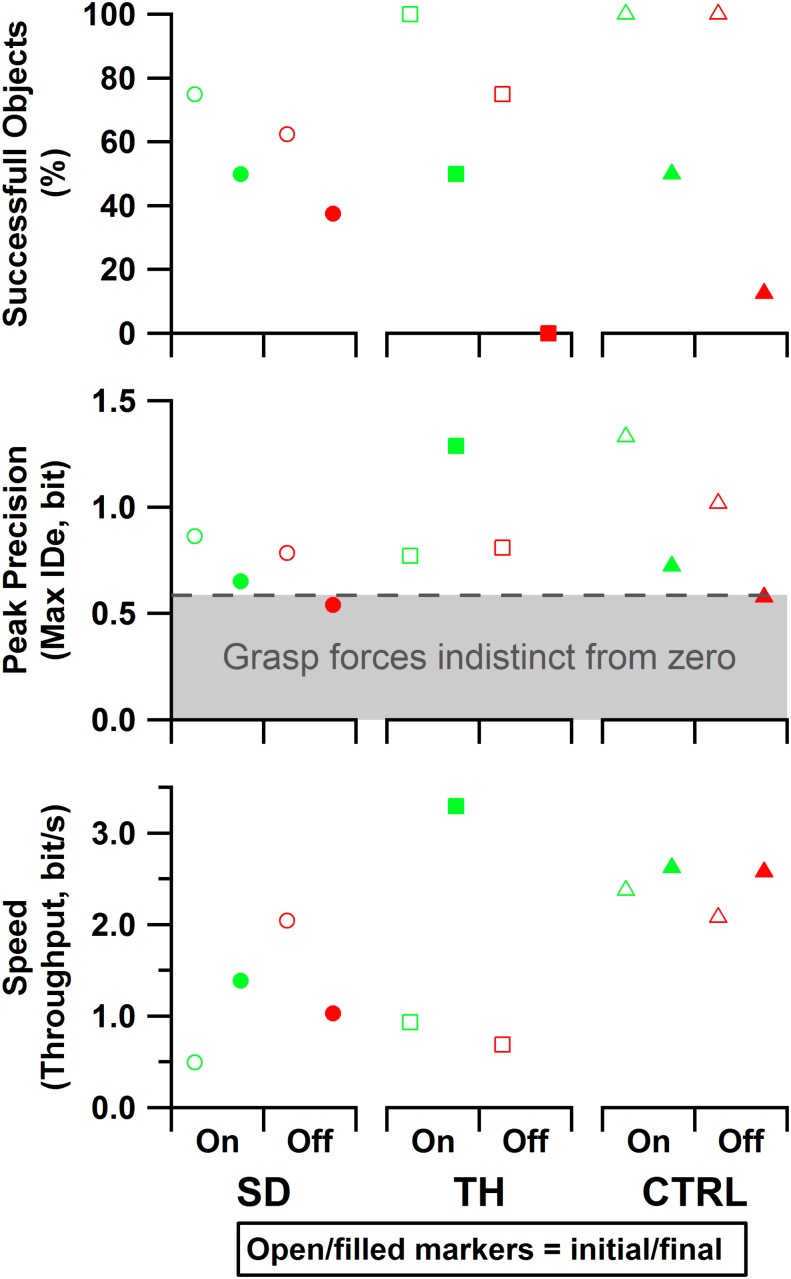
Results are shown for the participant with a shoulder disarticulation (SD, left column, circles), the participant with a transhumeral amputation (TH, center column, squares), and the control participant (CTRL, right column, triangles) in their visit before (open markers) and after (filled markers) the take-home period. Data presented are from the touch-on (green) and touch-off (red) conditions. The dashed line and shading indicate the area in which, statistically, there was not reliable grasp production (i.e., force greater than zero). Note that since TH’s initial visit did not include precision trials, the peak precision values for the initial visit may be slightly underestimated. Also note that since TH did not successfully handle any objects without touch feedback in the final visit, peak precision and throughput are undefined for that case.

For the initial visit, both SD and TH demonstrated tradeoffs in performance when given touch; SD achieved greater precision at the cost of speed, and TH showed increased speed and objects successfully handled at the cost of some precision (Figure 10). However, both participants successfully handled more objects with touch feedback. CTRL made an objective improvement when given touch, successfully handling objects with greater precision and faster speed. During the final visit, the benefits of touch were more pronounced, as both SD and CTRL improved in each of the three outcome measures. Additionally, providing touch allowed both participants to achieve a peak precision outside of the area in which, statistically, there was not reliable grasp production (i.e., force greater than zero). TH could only complete the task with touch during her final visit (zero successful objects without touch), thus touch provided a clear improvement. In all cases, providing touch improved at least two of the three outcome measures.

### Box and Block Task

**FIGURE 11:**
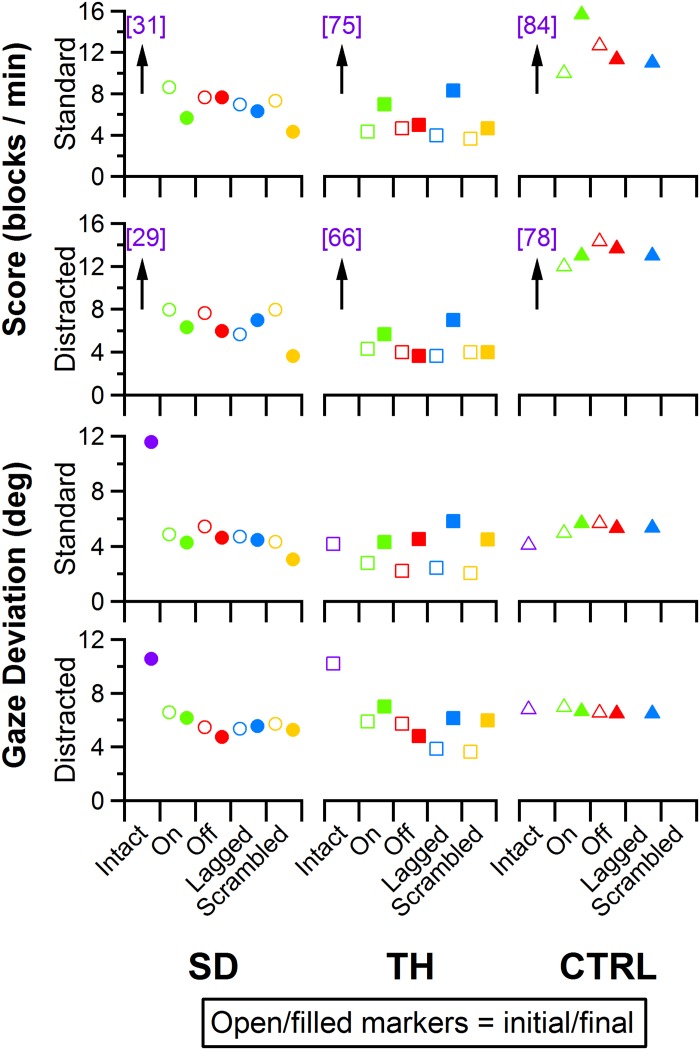
Score and gaze deviation during the Box and Block task are presented in the top two and bottom two rows, respectively. In both pairs, the top row shows the results during the standard task while the bottom row shows the results during the trials when a visual distractor was present. Results are shown for the participant with a shoulder disarticulation (SD, left column, circles), the participant with a transhumeral amputation (TH, center column, squares), and the control participant (CTRL, right column, triangles) in their visit before (open markers) and after (filled markers) the take-home period. Participants’ performance with their intact side are presented on the left (purple), followed by performance with the amputated side during the (from left to right) touch-on (green), touch-off (red), lagged (blue), and scrambled (yellow) conditions. Able-bodied scores that are outside of the *y*-axis range are shown in brackets.

Participant Box and Block scores were insensitive to the touch conditions presented (Figure 11, top two rows). Additionally, the presence of the visual distractor had little effect on Box and Block scores (Figure 11). Gaze deviation away from the prosthetic hand generally increased when the visual distractor was present (Figure 11, bottom row); touch conditions had a comparatively smaller effect on gaze deviation away from the prosthetic hand (Figure 11, bottom two rows). The most notable trend was that during the final visit, SD, and TH both looked at the prosthetic hand more during the touch-off condition (decreased gaze deviation), and were able to look away from the prosthetic hand more (increased gaze deviation) during the touch-on condition. Although gaze deviation was generally highest during the touch-on condition, gaze deviation tended to be greater when touch feedback was on, lagged, or scrambled than that in the touch-off condition.

### Clothespin Relocation Task

**FIGURE 12:**
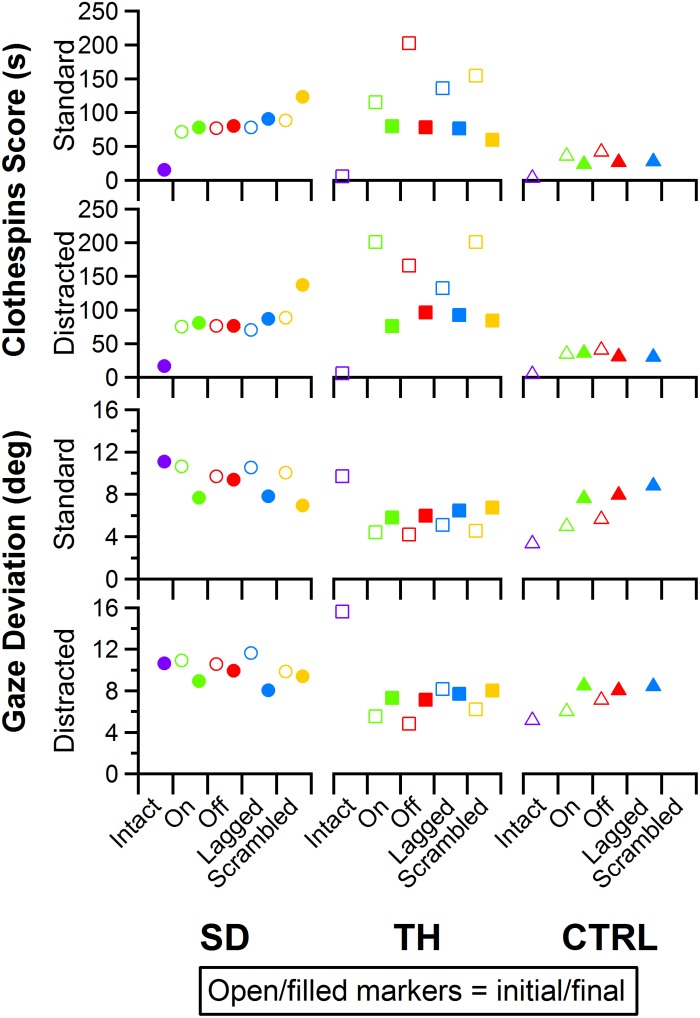
Score and gaze deviation during the Clothespin Relocation task are presented in the top two and bottom two rows, respectively. In both pairs, the top row shows the results during the standard task while the bottom row shows the results during the trials when a visual distractor was present. Results are shown for the participant with a shoulder disarticulation (SD, left column, circles), the participant with a transhumeral amputation (TH, center column, squares), and the control participant (CTRL, right column, triangles) in their visit before (open markers) and after (filled markers) the take-home period. Participants’ performance with their intact side are presented on the left (purple), followed by performance with the amputated side during the (from left to right) touch-on (green), touch-off (red), lagged (blue), and scrambled (yellow) conditions.

Performance in the Clothespin Relocation task was also mostly insensitive to the touch condition. SD demonstrated slower task completion times for the scrambled touch condition, especially during the final visit, whereas task completion times for the other touch conditions were relatively consistent during both visits (Figure 12, top two rows). During the initial visit, TH demonstrated the fastest task completion times in the touch-on condition. This effect was not present in the final visit. Rather, completion times were consistent except for the scrambled condition, when participants showed faster completion times. The addition of a visual distractor did not cause systematic changes in completion time across touch conditions (Figure 12). There were no systematic trends in eye gaze deviation relative to touch condition (Figure 12, bottom two rows); furthermore, the addition of a visual distractor had little effect on eye gaze deviation (Figure 12, bottom two rows). Clothespin drops (Supplementary Figure 3) were relatively low across all participants, visits, and touch conditions, with one or zero drops during the majority of test conditions. No systematic trends between initial and final visits were identified.

## Discussion

This work demonstrates that the relationship between users and sensate NMI prostheses is dynamic and changes over time with long-term use. Taken together, these results suggest that although the restoration of touch sensation can provide a near-immediate impact on operation of a prosthesis, long-term use may lead to further functional improvements, more appropriate integration of artificial limbs as a part of the body, and adaptation of higher-level neural-cortical systems.

### Neural Adaptation

The touch mapping experiments support that neural and cortical adaptation processes occurred with the long-term use of NMI prostheses that provided physiologically relevant touch feedback. It appears that continual exposure to, and use of, this restored sense of touch allowed the sensory architecture of the missing hand to conform to this new sensory information. Following the take-home period, CTRL, an NMI-prosthesis user who did not take home a touch-integrated device, demonstrated no significant changes in the proportion of skin producing missing hand percepts. In contrast, we found that the proportion of reinnervated skin producing sensations projected to the missing hand increased significantly for both SD and TH. The areas of the missing hand whose proportional representation most increased were often the same areas targeted by the touch tactors (ventral side of the five digits and distal palm). Not only did the proportion increase, but participants also reported feeling larger areas within these missing hand regions. These areas were also spatially congruent with the instrumentation installed on their prostheses. Inconsistent or minimal increases were observed in the missing hand areas that were not directly targeted by participants’ touch feedback systems. Across our participants, little to no growth was observed in the proximal palms (near the wrist) and minimal increases in proportion of projected sensation were observed in the dorsal side of the hands.

The implementation of sensory feedback within the constraints of a prosthetic fitting is complex. There are a number of functional constraints that must be considered. Foremost is the limited surface area available for both touch feedback tactors and EMG-control electrode placement. Here, a balance must be struck between the available touch percepts on the skin and the available motor control points in the reinnervated muscle. SD is an excellent example of this. We focused primarily on the touch feedback system targeting the most functionally relevant digits for the use of a three-jaw-chuck myoelectric terminal device (thumb and index finger). Multiple strong thumb and index finger touch percepts were available for SD, so we identified locations where reported sensation was most congruent to sensor locations on the prosthesis. We also set the pressure and tap settings (gains) for those two tactors to follow what the participant suggested ‘felt most correct’ (Figure 3). The remaining tactors were placed in areas that most closely approximated the palm and middle, ring, and little fingers. These positions were under more constraints with respect to placement on the remaining socket and skin areas. At these locations, the pressure and tap gains were set to provide reliable touch sensation to minimize the possibility of interference with the EMG control, either by electrical crosstalk or by displacing the skin that was in contact with the control electrodes.

We found changes in SD’s final percept map that appeared to reflect both physical aspects of the prosthesis and the spatial accommodations made for the sensory feedback and control systems. For example, we found that the thumb and index finger appeared to blend together. This may be an effect of the coupling between the main digits on three-jaw-chuck myoelectric hands, where digits 1–3 (thumb, index finger, and middle finger) always operate together. Moreover, the proportional and tap gains were higher, which made the thumb and index finger more sensitive. In the final hand map, we saw a focus on the thumb and index finger without the addition of the coupled middle finger. Similarly, the ring and little fingers also appeared to blend together in the final percept map. Both of these digits are passive in the myoelectric hand and are physically coupled together by an internal wire frame. In contrast, the separate palm tactor appeared to remain largely focused toward palm percepts. More broadly, we found an expansion of the thumb/index-finger and ring/little finger percepts across the final touch map. The thumb and index finger demonstrated the most expansion across the mapping space. Similarly, the ring-/little-finger representations also expanded; however, this effect was less pronounced than the thumb/index-finger changes. There is a possibility that the expansion was related to the roughly 1.5 cm lateral shifting of the tactor heads (Figure 3). This shifting was related to body shape changes experienced by SD over the duration of the take-home period. Although socket fit was impacted, the thumb- and index-finger-focused expansion also extended medially on the final touch map and the relatively small position shift did not impact EMG function.

Although the changes in TH’s final percept map were different than SD’s, they appear to be driven by the sensory-motor constraints and accommodations unique to her prosthesis fitting. Unlike SD, whose socket rested against her chest and allowed tactors to press directly on the chest skin (see: Figures 1, 3), TH used a silicone liner between the residual limb and the hard plastic socket where the tactors were mounted. In this setup the tactors could not touch the skin directly. They instead pressed into the reinnervated skin through the liner, which had thinned-out sections to improve sensation while maintaining traction and socket suspension on the residual limb. Furthermore, TH used a pattern recognition system for controlling her prosthesis, which is not sensitive to electrode positioning. In TH we saw a widespread increase in the strength of the touch percepts projected to the missing hand. We also saw an expansion of the percept areas that correspond to the digit and palm placement of the tactors. However, instead of focusing the percepts in the final touch map, we saw a fusing of multiple digits, similar to SD. Although this may have involved the coupling of the three-jaw-chuck myoelectric fingers, it is possible that other factors related to the prosthetic fitting also influenced the perceptual changes. Since TH’s tactors had to press through a liner to reach the reinnervated skin, the tactor influence may not have been as locally focused as it was for SD. Instead, each time the tactor pushed in on a specific spot, the surrounding reinnervated skin may have experienced indirect stimulation. This may have activated other local touch percepts simultaneously. Also, from a practical perspective, since TH wore a socket liner, there may have been minor changes in position each time it was donned. Changes in position due to donning compounded with activation of adjacent percepts due to pushing through the liner likely account for the “smearing” of tactor-elicited sensations in TH’s final touch map.

Although we cannot rule out that changes to the sensory reinnervation of the skin itself may have occurred due to repeated stimulation over the course of the take-home period, our data suggest that brain processing may be impacted by the long-term restoration of physiologically relevant touch. Psychophysical temporal order judgment tasks can be used to study the interplay between different senses, such as vision, hearing, and touch (Keetels and Vroomen, 2012). PSS is a time-based evaluation of perceptual shifts, derived from responses in a temporal order judgment task, that implicitly measures weighting given by the brain to different sensory information channels (Moseley et al., 2008). PSS occurs when two streams of sensory information are perceived as occurring at the same time. Therefore, when two sensory channels receive equal weighting they will yield a PSS of 0, while a larger magnitude correlates to greater differences in weightings (Vroomen et al., 2004). Similar to Marasco et al. (2011), we applied PSS measures to investigate the equivalency of sensory processing from the amputated side relative to the intact side, and how the brain may adapt its weightings with long-term restored touch sensation. We used an experimental paradigm that applied equivalent vibratory stimuli to non-reinnervated skin at mirrored positions on each participant’s limbs to probe central tactile processing mechanisms. Before the take-home period, when two equivalent vibratory stimuli were provided at the exact same time, we found that all the participants were more likely to identify stimuli on the amputated side as occurring first. This suggests the brain weighted the sensory information from the amputated side asymmetrically from the intact side (regardless of the feedback condition: touch-off, touch-on, lagged, scrambled, or fixation). In contrast, following the take-home period, SD and TH demonstrated more symmetric PSS results regardless of feedback conditions. Before and after the take-home period CTRL remained largely unchanged. These findings support the idea that the brain changed its processing of sensory information and became more comparable to that of the intact side. Of particular relevance, in the touch-off and fixation conditions, the peripheral sensory receptors associated with the missing hand were not receiving any touch feedback stimulation; yet, PSS measures were more symmetric. The persistence of increased symmetry without touch feedback supports the idea that these changes were occurring above the peripheral neural level. Additionally, since results remained relatively consistent across feedback conditions within a test session, this symmetry may be a longer-term result of the brain adapting to the returned touch sensation of the amputated side rather than an immediate system response.

The sense of limb ownership arises from the integration of visual and tactile information; that is, when a stimulus is seen and felt appropriately (temporally and spatially congruent), the brain assumes ownership over that part of the body (Botvinick and Cohen, 1998). In the initial embodiment experiments, SD and TH demonstrated a tendency to embody their prostheses under conditions where touch was either temporally (lagged) or spatially (scrambled) mismatched in addition to the normal temporally and spatially appropriate (touch-on) condition (Figure 8). After the take-home period, this tendency was abolished – SD and TH only showed embodiment during the touch-on condition, where all multisensory temporal and spatial inputs were appropriately aligned. The initial tendency of the participants to take ownership of the prosthesis in conditions with mismatched touch indicates abnormal brain processing that is overly permissive when establishing body ownership. This permissiveness in ownership can also be seen in individuals who inappropriately experience the pain of others as their own, or experience touch when they see others being touched (Aimola Davies and White, 2013; Botan et al., 2018). This condition arises when the brain incorrectly assumes ownership of external features due to simple correlations of multisensory information, despite the temporal and/or spatial relationships between sensory channels being inappropriate. Similar to these populations, SD and TH also integrated inappropriate yet correlated information to establish embodiment during their initial visits. In the lagged condition, participants received touch input from the tactors 1000 ms after seeing the actual touch by the investigator on the prosthesis. Although the timing between what was seen and what was felt was shifted, the two sensory events were still correlated. Similarly, in the scrambled condition, the touch tactors were connected to mismatched sites on the reinnervated skin (e.g., when the investigator touched the thumb of the prosthesis, the participant felt touch on a different digit). Although touch on the prosthesis was spatially mismatched with touch felt on the missing hand, the timing was correlated. The prolonged return of sensation matched to relevant prosthesis activity during the take-home period appears to have provided the contextual cues necessary for restoring these individuals to a more normal mode of multisensory processing (Heed and Azañón, 2014). Interestingly, previous work with one of the participants shows that their prosthetic limb was not embodied when there was a complete lack of temporal correlation between touch and vision when touches experienced by the participant through the touch tactor system were randomly associated with observed touches to the prosthetic hand (Marasco et al., 2011). Furthermore there appears to be a limit to the cortical-representational distance that can lead to permissiveness in body attribution. In the earlier study discussed above, there was a similar temporal correlation between mismatched spatial locations, yet the participant did not embody the prosthesis when touch seen on the prosthesis forearm was felt on the missing hand (Marasco et al., 2011), which was representationally located much farther away than the mismatched locations in the study reported here. In the present study, during the initial visits, the incorrectly correlated input from different digits in the scrambled condition promoted overly permissive embodiment. Long-term use of a touch-integrated prosthesis likely helped build a stronger perceptual representation of the fingers, which contributed to a less permissive yet more normal mode of limb ownership.

Participants performed this study using a single-degree-of-freedom prosthetic hand that provided a three-jaw-chuck grasp configuration. However, there are numerous dexterous prostheses available that can perform multiple hand grasping configurations. With these devices, not all digits are involved in each possible grasp configuration and each configuration is visually distinguishable from the next. Therefore, in more dexterous systems, the impact of spatial congruency in what is seen and felt is likely to be even more important than with a single-degree-of-freedom hand. As prostheses become increasingly sophisticated, the quality and congruency of sensory feedback will progressively become more important to integrating these systems as a true limb replacement.

### Functional Changes

Our block-foraging and psychophysical grasp tasks are standardized, scientifically validated tests that, when applied to upper-limb prosthesis use, can parse out the contributions of control and sensory feedback in relation to successful task completion (Thumser et al., 2018; Beckler et al., 2019). We found that across all three participants, there appeared to be an immediate improvement in functional performance when touch was turned on. Interestingly, SD’s and TH’s functional performance following the take-home period remained near the initial levels and did not improve substantially over that.

Our block-foraging task examines sensory discrimination and evaluates how sensation influences discrimination strategies and decision-making. Across all participants both before and after the take-home period, when provided with touch feedback, trials took longer. This test isolates cutaneous force as the primary sensory channel informing participant decision-making. When no touch feedback is present, prosthesis control is open-loop and participants have no sensory information outside of vision on which to base decisions. However, when touch is turned on, participants slow down as they engage with the sensory information to make more careful, informed decisions when searching for blocks of a target stiffness. The addition of this sensory channel improved the accuracy of selecting a correct target block; however, the likelihood of interrogating a correct block but not selecting it also increased (false negative rates). With touch feedback, participants were willing to interrogate more blocks and improve accuracy at the cost of speed. Handling time is a measure of one’s ability to pilot the prosthesis, and it generally did not change in relation to touch feedback. This suggests that the changes in search time were primarily due to interaction with sensory feedback. Following the take-home period, the initial improvements in accuracy with touch feedback remained. These results provide evidence that the cutaneous force information provided through the NMI is readily used by the brain with little learning required.

Our psychophysical grasp task is designed to evaluate how quickly and precisely individuals can reach their intended grasping force. Similar to results found with our block-foraging task, turning on the touch feedback system resulted in immediate changes in task performance across all three participants. Touch enabled all participants to more reliably achieve intermediate grasp forces. However, in their initial visits, both SD and TH had to make compromises, either improving grasp force precision at the cost of speed (SD) or improving speed at the cost of precision (TH), whereas CTRL improved both speed and precision. In contrast, following the take-home period, both SD and TH were able to use the sensation of touch to improve both speed and precision. Similar to conclusions drawn from the block-foraging task results, it appears that initially the sensory information provided through the NMI was readily interpreted by the brain, and the use of the touch system during the take-home period provided the brain with additional context for the sensory information. This appears to have helped the users integrate their restored sense of touch into their prosthesis control strategies. For example, TH was unable to complete the psychophysical grasp task without touch in her final visit, indicating that touch feedback had become an essential part of her control strategy and without it, achieving intermediate grasping forces was extremely difficult. These results suggest that long-term use of NMI touch feedback can promote more effective closed-loop control that enables users to more quickly and precisely achieve intended grasping forces.

We observed significant changes in function, as well as cognition and perception, in response to touch sensation across multiple measures; however, these changes were not represented in the Box and Block or Clothespin Relocation tasks. We attempted to capture changes caused by touch feedback by adding eye gaze tracking and trials with a distractor video to the Box and Block and Clothespin Relocation tasks. Our intent was to use the eye gaze data as a proxy to capture visual attention during the task. We found that for the Box and Block task the participants demonstrated slightly more gaze deviation in the touch-on condition. However, this result did not translate to the Clothespin Relocation task. Relocating a clothespin requires a higher amount of visual attention to pinch, rotate, transfer, and release an object in a precise location. Here, there is little opportunity for gaze deviation from the hand, which is reflected in the results. Eye tracking data may provide complementary information to quantify the attention and visual demand when operating a sensate prosthesis; however, tasks must be carefully designed to permit looking away from the prosthesis and/or require decisions based on sensory feedback to reflect the true impact of this sensation.

Integrating a prosthesis as a functional body part is a complex challenge. Although sensation fundamentally underpins body perception and limb function, its contributions are difficult to quantify as its influence is multifaceted and dependent on many factors (Markovic et al., 2018). For example, we found minimal performance difference with and without touch sensation in the standard clinical measures of Box and Block and Clothespin Relocation tasks. We argue that these tasks did not sufficiently challenge users to engage with touch sensation to shape task behaviors. Rather, visual cues and motor control drove task performance. The block-foraging and psychophysical grasp tasks were specifically designed to require that participants engage with touch feedback (Thumser et al., 2018; Beckler et al., 2019). In these tasks, participants displayed a near-immediate improvement in performance when touch sensation was provided; however, no additional improvements were seen after the take-home period. This lack of additional long-term improvement may be attributed to the touch feedback system utilizing the residual neural anatomy associated with the now-missing hand. Following amputation, the brain retains a representation of the missing hand and is likely able to readily use sensations generated through this residual architecture with minimal learning. This is because the restored touch information is felt as an equivalent touch in the missing hand. In the same way that we would anticipate minimal performance changes if a healthy, intact hand were tested before and after a 2-year span, our participant cohort performed similarly on functional tasks over a comparable time span. It was only through the employed cognitive and perceptual measures that the long-term effects of restored touch became evident. Here, we employed multiple measures to better understand the relationship between the newly restored sense of touch and higher-level sensory processing. Across multiple independent measures, we found evidence to suggest neural-cognitive adaptation processes occur with the long-term use of NMI prostheses. Therefore, we argue that evaluation of sensate prostheses must extend beyond functional tasks and performance measures to further capture the integration of the system as a part of the body. Quantifying this process is complex and requires multiple independent measures to capture changes in functional abilities, the user’s explicit perceptions that the device is a body part, and the implicit processes in which sensory-motor mechanisms adapt and are learned.

## Conclusion

Restoring touch sensation through NMI prostheses brings us one step closer to true limb replacement. However, achieving this goal will require a paradigm shift in the way we study and evaluate advanced robotic limbs. Rather than viewing prostheses as tools used to improve function, we must begin evaluating these devices as integrated body parts. Future investigations following the development and growth of this dynamic relationship over the long term are important next steps in this exciting process of unlocking the next generation of integrated artificial limbs.

## Data Availability Statement

The data sets generated for this study are available upon reasonable request to the corresponding author.

## Ethics Statement

The studies involving human participants were reviewed and approved by the Cleveland Clinic Institutional Review Board and the Department of the Navy Human Research Protection Program. The patients/participants provided their written informed consent to participate in this study. Written informed consent was obtained from the individual(s) for the publication of any potentially identifiable images or data included in this article.

## Author Contributions

All authors contributed to the manuscript preparation. PM, ZT, and DB contributed to the experimental design and development, and fabrication of the experimental prostheses. ZT, DB, CS, and JS performed the experimental data collection and data analysis. PM and CS coordinated with participants. PM was the principal investigator, provided scientific direction, and performed overall project coordination. ZT, CS, and DB performed technical support for participants during the take-home period.

## Conflict of Interest

The authors declare that the research was conducted in the absence of any commercial or financial relationships that could be construed as a potential conflict of interest.
