# Supplementary material for: Long-Term Home-Use of Sensory-Motor-Integrated Bidirectional Bionic Prosthetic Arms Promotes Functional, Perceptual, and Cognitive Changes
Source: Front Neurosci. 2020 Feb 19;14:120. doi: 10.3389/fnins.2020.00120 (PMC7042391; doi:10.3389/fnins.2020.00120)
Supplement: Supplementary file 1 [file Table_1.docx]

***Supplementary Material***

# Supplementary Figures
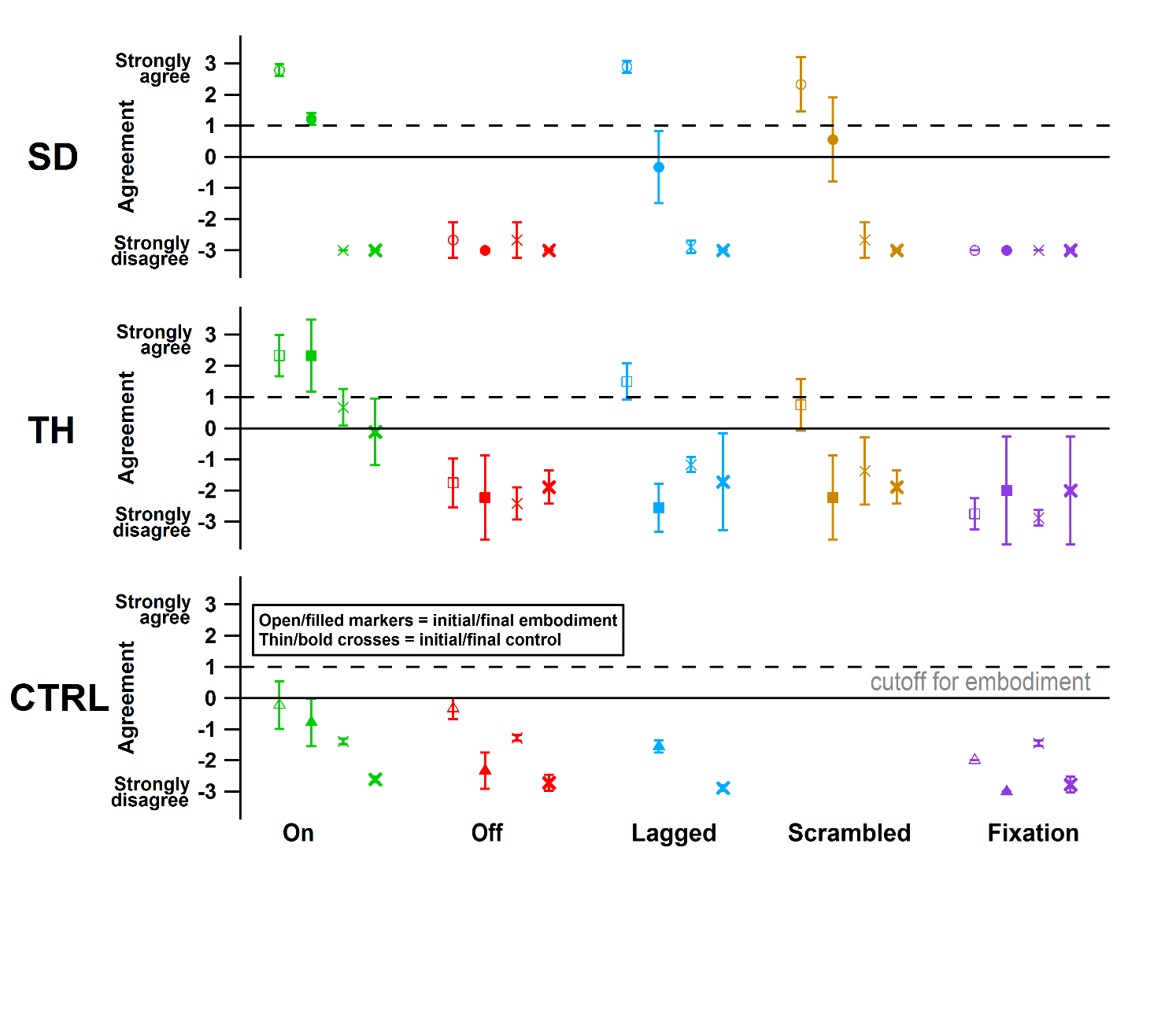


**Supplementary Figure 1.** Complete embodiment questionnaire data set. This figure includes all the information shown in Figure 8 with the addition of the control statement results. The degree of agreement with questionnaire statements is shown for the participant with a shoulder disarticulation (SD, top row, circles), the participant with a transhumeral amputation (TH, center row, squares), and the control participant (CTRL, bottom row, triangles) in their visit before (open markers) and after (filled markers) the take-home period. Responses to control questions are shown as crosses, with thin and bold line weights indicating the responses during the initial and final visit, respectively. Markers are colored according to touch feedback condition: touch-on (green), touch-off (red), lagged (blue), scrambled (yellow), and fixation (purple). All participants responded below the cutoff for agreement to the control questions (greater than or equal to one (Kalckert and Ehrsson, 2012), indicated by the dashed line).

**
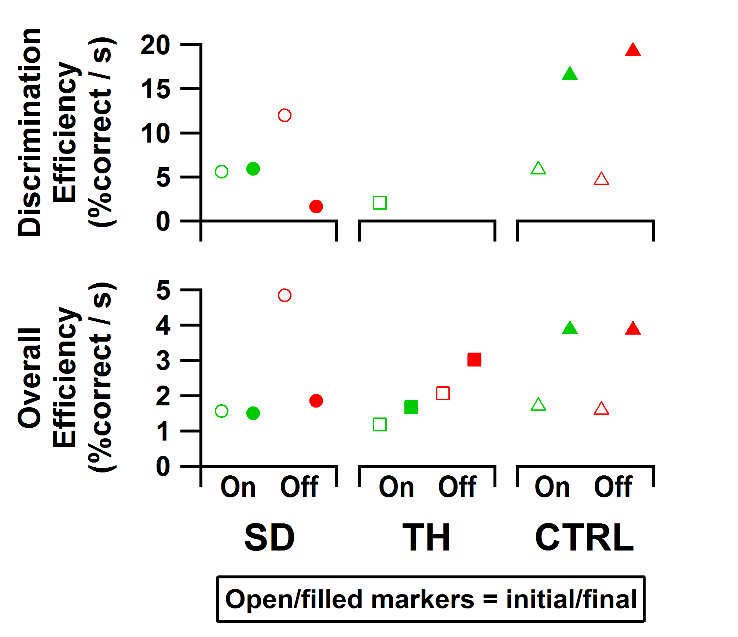
**

**Supplementary Figure 2.** Discrimination efficiency and overall efficiency data are presented for the participant with a shoulder disarticulation (SD, left column, circles), the participant with a transhumeral amputation (TH, center column, squares), and the control participant (CTRL, right column, triangles) in their visit before (open markers) and after (filled markers) the take-home period. Data are presented for the touch-on (green) and touch-off (red) conditions. Note that TH received discrimination efficiency scores of zero during each test due to her inability to discriminate above chance, except for the touch-on condition during the initial visit.


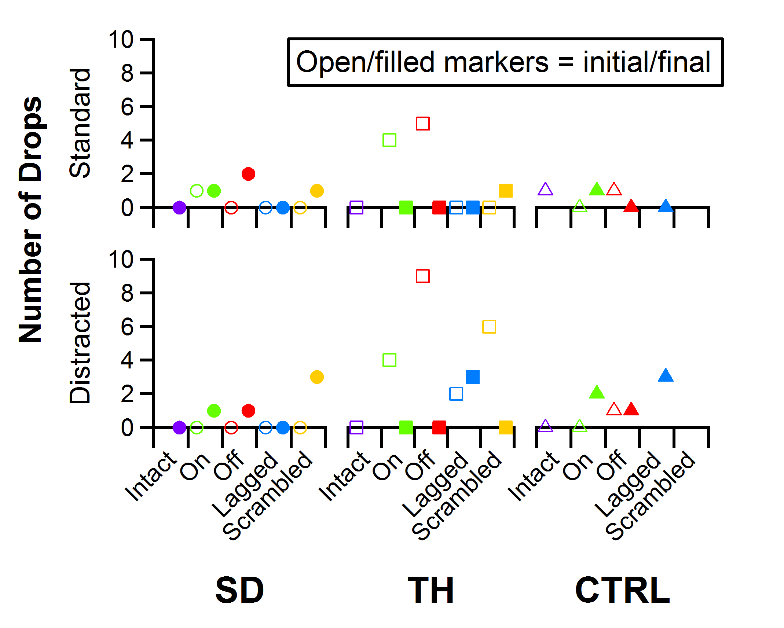


**Supplementary Figure 3.** Number of drops during the Clothespin Relocation task are presented. The top row shows the results during the standard task while the bottom row shows the results during the trials when a visual distractor was present. Results are shown for the participant with a shoulder disarticulation (SD, left column, circles), the participant with a transhumeral amputation (TH, center column, squares), and the control participant (CTRL, right column, triangles) in their visit before (open markers) and after (filled markers) the take-home period. Participants’ performance with their intact side are presented on the left (purple), followed by performance with the amputated side during the (from left to right) touch-on (green), touch-off (red), lagged (blue), and scrambled (yellow) conditions.
